# Lifestyle factors affecting the pathogenesis of androgenetic alopecia: a literature review

**DOI:** 10.3389/fpubh.2026.1739298

**Published:** 2026-02-04

**Authors:** Fujun Huang, Lei Tang, Xun Zhou, Qiang Fu, Yiping Lu

**Affiliations:** 1 Liaoning University of Traditional Chinese Medicine, Liaoning, Shenyang, China; 2 Department of Cosmetic Dermatology, Chongqing Traditional Chinese Medicine Hospital, Chongqing, China; 3 Chengdu University of Traditional Chinese Medicine, Sichuan, Chengdu, China

**Keywords:** androgenetic alopecia, inflammation, lifestyle, oxidative stress, pathogenesis

## Abstract

Androgenetic alopecia (AGA), which significantly impairs patients’ social interactions and psychological well-being, is widespread worldwide. Treatment of AGA is a long-term process that is difficult to stick to. Therefore, in the long-term management of AGA, establishing effective lifestyle intervention protocols to delay disease progression has become a central focus for both clinicians and patients. However, to the best of our knowledge, only limited and fragmented studies have characterized the impact of lifestyles on AGA. In this review, we focused on the impact of lifestyle factors, such as dietary habits, sleep patterns, ultraviolet radiation, exercise, and hairstyles, on AGA, and examined the underlying pathogenic mechanisms by which these factors may induce or exacerbate AGA.

## 1 Introduction

Androgenetic alopecia (AGA) is a progressive hair loss disorder that primarily affects adolescents and post-adolescents and is characterized by the miniaturization of hair follicles (HFs). It is the most prevalent form of alopecia in humans. The prevalence of this disease increases with age, with up to 42% of females and 80% of males aged ≥ 70 years exhibiting the characteristic manifestations of AGA (1). However, its clinical manifestations can be traced back to the prepubertal stages, with an average age of onset of 12.9 ± 2.7 years (2). Skin is the outermost layer of the body, constantly and inevitably exposed to multifarious environmental conditions (3). HFs, as a vital component of the scalp, are predominantly located in the vertex region and are susceptible to external environmental factors such as ultraviolet radiation (UVR), mechanical traction, and chemical agents like hair dye (4–7). HFs’ physiological functions are significantly correlated with hormonal regulation and immune cell activity (4). AGA is a representative disease of HFs. Male patients typically present with frontal, temporal, and/or vertex hairline recession, which progresses to diffuse thinning and eventual scalp exposure. The primary clinical manifestation in female patients is diffuse thinning and hair shaft miniaturization on the vertex and scalp partition line, with retention of the frontal hairline (8). It often severely impairs patients’ social interactions and psychological well-being (9, 10). However, current mainstream therapeutic modalities fail to simultaneously address efficacy, safety, comfort, and cost-effectiveness: ① Oral pharmacotherapy for treating AGA, such as finasteride, dutasteride, and spironolactone, involves prolonged treatment durations and may cause adverse effects, including androgenic sexual dysfunction in males and menstrual irregularities in females (11, 12). ② Topical/Oral minoxidil is associated with adverse effects like the transient shedding and hypertrichosis. Furthermore, discontinuation often precipitates the recurrence of AGA (13, 14). ③ Physical interventions such as hair transplantation, laser therapy, and platelet-rich plasma (PRP) treatment are costly and uncomfortable (15). These problems lead to patient’ poor compliance, interrupted treatment course, and suboptimal therapeutic effects.

Therefore, in the long-term management of AGA, establishing effective lifestyle intervention protocols to delay disease progression has become a central focus for both clinicians and patients. To the best of our knowledge, only limited and fragmented studies have characterized the impact of lifestyles on AGA. The article outlined the specific role of lifestyle factors in AGA. Furthermore, the specific pathological mechanisms by which these lifestyles cause or exacerbate AGA have been investigated, based on the pathophysiological mechanisms of AGA.

## 2 Lifestyle factors associated with AGA

**Figure 1:**
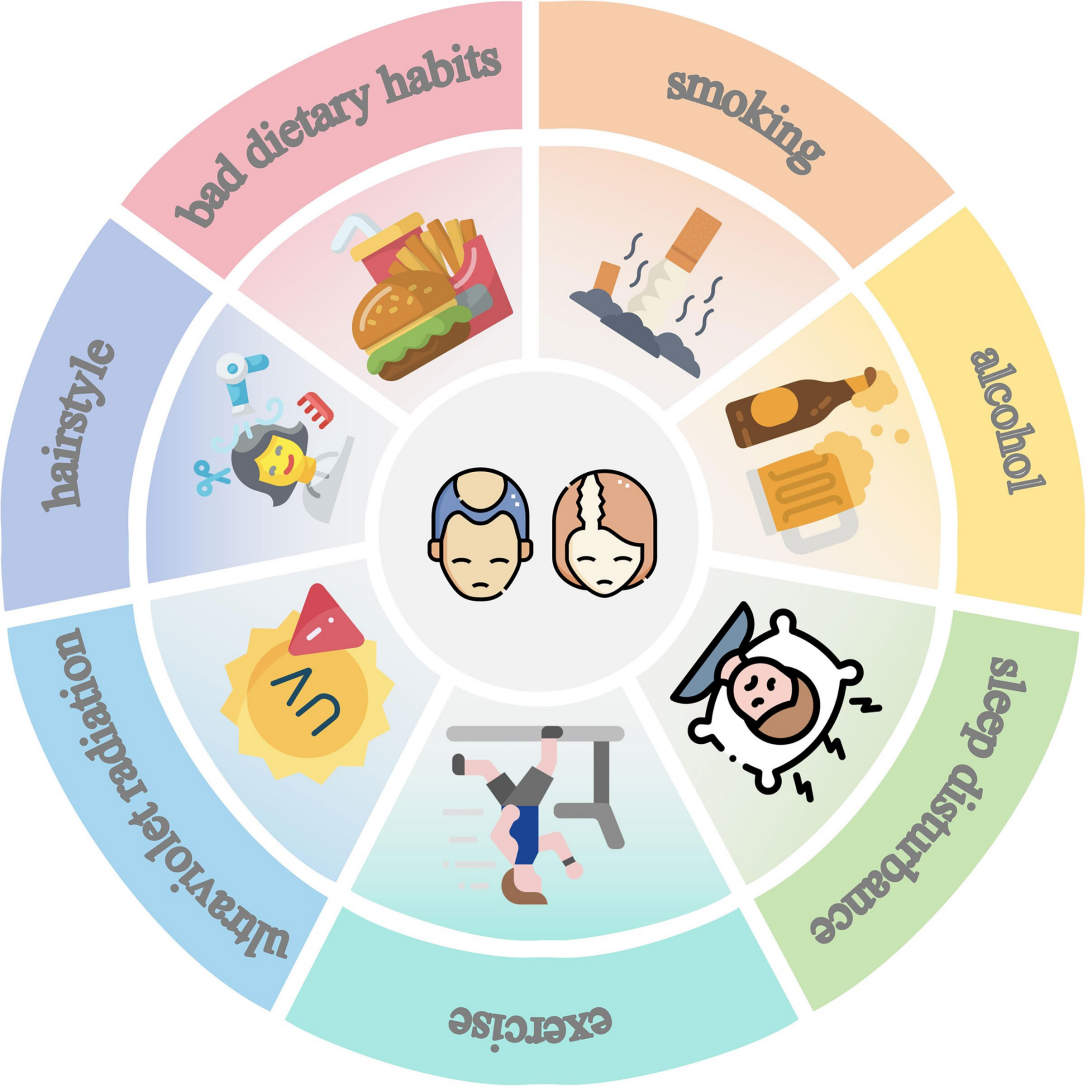
Lifestyle of AGA. The figure has been designed using resources from Flaticon.com.

Numerous lifestyle factors, such as dietary habits, sleep patterns, exercise, and UVR, may be closely associated with the development of various diseases, including AGA (Figure 1).

### 2.1 Dietary habits

#### 2.1.1 High-fat diets

High-fat diets cause overeating and weight gain, suggesting that they are sufficient to cause obesity (16): ① Kim S. et al. (17) indicated that obesity is associated with elevated free testosterone. Insulin resistance, often induced by obesity, is the underlying abnormality for high insulin levels (18). Ovarian and adrenal glands exhibit increased androgen synthesis, such as testosterone (which can indirectly lead to the miniaturization of hair follicles), when exposed to high concentrations of insulin (19). Meanwhile, Xie B. et al. also confirmed that the androgen levels in patients with AGA were higher than those in normal individuals (20). ② Microcirculation of the scalp may be impaired by hyperlipidemia, resulting in a reduction of blood flow in the capillary vessels of HFs, and the hair follicle is subsequently deprived of sufficient nutrition (21). ③ Adipose tissue in obese patients exhibits hypersecretion of a spectrum of proinflammatory cytokines, including monocyte chemoattractant protein-1 (MCP-1), tumor necrosis factor-alpha (TNF-α), interleukin-1 beta (IL-1β), interleukin-6 (IL-6) (22), etc. The pro-inflammatory cytokines can inhibit growth of HFs (23, 24). ④ Obesity can provoke oxidative stress, which ultimately manifests as arrested hair growth and accelerated alopecia, by altering the adipose microenvironment (adipocytes, macrophages), facilitating low-grade chronic inflammation, and causing mitochondrial malfunction (mitochondrial division, fusion) (25–27). ⑤ The hyperactivity of sebaceous glands frequently observed in obese patients causes excessive sebum accumulation on the scalp, disrupts the local microbial homeostasis, and promotes the proliferation of *Malassezia* (28, 29). *Malassezia* contributes to the pathogenesis of AGA through multiple mechanisms (30). ⑥ Wang S. et al. (31) observed that obese patients frequently exhibit downregulation of peroxisome proliferator-activated receptor gamma (PPARγ). One of the ligand-dependent transcription factors for the nuclear receptor superfamily is PPARγ (32), which can inhibit oxidative stress, downregulate inflammatory pathways, and reduce androgen receptor (AR) transcriptional activity (33, 34). The loss of PPARγ in hair follicle stem cells (HFSCs) may promote the occurrence or progression of AGA.

#### 2.1.2 Deprivation (crash) diets

Deprivation (crash) diets can have a significant negative impact on the endocrinological balance of the human body (21): ① Colling C. et al. (35) found that there was an increase in free cortisol and total cortisol after a 10-day high-calorie and fasting protocol. Elevated levels of cortisol can induce or exacerbate AGA by suppressing activity of HFSCs and/or accelerating the premature transition of HFs into the catagen phase (36, 37). ② Starvation diets often result in malnutrition, which can lead to deficiencies in essential micronutrients (such as zinc, magnesium, iron, and vitamins A and D) critical for proliferation of HFs (38). It can directly impair the ability of hair matrix cells to produce healthy hair (39).

#### 2.1.3 Caffeine diets

One of the important food additives found in beverages and foods is caffeine (40). Ly N. et al. (41) reported that caffeine appears to be a safe and promising potential treatment for hair loss: ① Testosterone-induced inhibition of hair follicle (HF) growth was reversed when applied in the lowest investigated concentrations of caffeine (10 and 50 μg/mL) (40). Its mechanism of action is believed to be that caffeine can inhibit the activity of 5-alpha reductase (5α-R), improve local microcirculation in the scalp skin, enhance hair shaft elongation, and stimulate hair matrix keratinocyte proliferation (42–44). In contrast, higher concentrations of caffeine (100, 500, and 1,500 μg/mL) were observed to have inhibitory effects on HF growth (40). ② Caffeine has often been proposed as an antioxidant agent (45). An improvement in oxidative stress may be accompanied by a corresponding alleviation of hair loss symptoms. ③ Fischer T. et al. (46) observed that caffeine can antagonize corticotropin-releasing hormone (CRH)-mediated stress in these HFs effectively counteract stress-induced hair damage and possibly prevent stress-induced hair loss.

### 2.2 Smoking

The diverse chemical constituents of tobacco are implicated in the pathogenesis of multiple inflammatory dermatoses (47), encompassing AGA (48): ① In humans, nicotine, the primary alkaloid present in tobacco, can be incorporated into hair shafts, whether through systemic absorption into the bloodstream or from direct exposure to environmental smoke (49). Scalp capillary vasoconstriction (leading to long-term malnutrition and hypoxia of HFs and impeding the regeneration of healthy hair) is induced by nicotine through impairing acetylcholine-induced endothelium-dependent cutaneous vasodilation (50, 51). ② Exposure to nicotine and other chemical toxins in tobacco can readily cause persistent inflammatory cell infiltration around HFs, damage nuclear deoxyribonucleic acid (DNA) and mitochondrial DNA in HFSCs and induce oxidative stress (52–56), thereby potentially contributing, in part, to HF miniaturization. ③ Guo L. et al. (57) discovered that cigarette smoke exposure decreased the activity of the Wnt/β-catenin signaling pathway, which is closely related to hair growth.

### 2.3 Alcohol consumption

It was found in Yang W. et al.’s research (58) that the incidence of AGA is higher among alcohol consumers than among abstainers: ① Alcohol consumption trended toward positive genetic correlation with total testosterone in males (59). Elevated testosterone is often accompanied by the occurrence of AGA (20). ② Chronic alcohol consumption may downregulate the Wnt/β-catenin pathway (60) and promote bone morphogenetic protein (BMP) synthesis (61). Activation of the BMP pathway induces irreversible outcomes, including quiescence of HFSCs, shortened anagen phase, and follicular miniaturization, by persistently trans mitting pathological signals that halt growth or by antagonizing the Wnt/β-catenin pathway and directly inhibiting the normal hair cycle (62). ③ Among the known members of the prostaglandin (PG) family, both prostaglandin E2 (PGE2) and F2α (PGF2α) possess hair growth-promoting properties and are indispensable substances for maintaining a healthy hair growth cycle (63). The diminished production of PGE2 and PGF2α resulting from chronic alcohol consumption (64) has a potential detrimental effect on the health of HFs. ④ Almost all alcohol (ethanol) in the human body is metabolized in the liver by alcohol dehydrogenase (ADH) to acetaldehyde, which is subsequently metabolized by aldehyde dehydrogenase (ALDH) to acetic acid. Ethanol and acetaldehyde can generate substantial oxygen species (ROS) and pro-inflammatory factors during metabolic processes (65, 66), thus providing a mechanistic basis that could lead to the suppression of hair growth.

### 2.4 Sleep disturbance

Sleep is an essential physiological function that accounts for one-third of human daily activity. Sleep disturbance can adversely affect human health and may induce or exacerbate AGA (67): ① Sleep deprivation directly disrupts the secretion of multiple hormones critically involved in the hair growth circle. Melatonin is predominantly synthesized in the pineal gland in mammals, with its synthesis and secretion confined to the nocturnal period (68). Zhang Y. et al. (69) believed that melatonin-mediated circadian signals exert a crucial regulatory effect on the state of HFSCs. Owing to the fact that it is not only a sleep hormone but also a potent antioxidant and hair growth regulator that directly stimulates the proliferation of keratinocytes (KCs) of HFs and prolongs the hair growth period (70). Sleep deprivation directly results in decreased melatonin secretion, depriving the essential protective and supportive factor of HFs. ② Cortisol is the end product and a crucial functional biomarker of the hypothalamic–pituitary–adrenal (HPA) axis, exhibiting a circadian rhythm with nadir levels observed around midnight (71). Sleep deprivation leads to increased cortisol levels (72), which may initiate or worsen AGA by inhibiting HFSCs function or hastening the transition of HFs into the catagen phase (37). ③ Extended sleep deprivation may disrupt the equilibrium of androgens and estrogens (73), potentially contributing to an environment that favors the aggravation of AGA (74). ④ One potential function of sleep is to mitigate oxidative stress within the central nervous system and peripheral tissues, facilitating the clearance of reactive metabolites (such as ROS) that accumulated during wakefulness (75). Sleep deprivation probably induces apoptosis in HF-associated cells due to oxidative stress resulting from excessive accumulation of ROS (76, 77). ⑤ A prevalent vicious cycle exists between sleep deprivation and psychological stress, wherein sleep deprivation induces psychological stress, which in turn further disrupts sleep architecture (78). Psychological stress impairs the transition of HFs from the telogen to the anagen phase via activation of the sympathetic nervous system/norepinephrine (NE)/cyclic adenosine monophosphate (cAMP) signaling pathway (79), thereby potentially contributing to the exacerbation of AGA.

### 2.5 Exercise

In recent years, with the advancement of nationwide fitness initiatives, clinical consultations regarding optimal exercise modalities and intensities for patients with AGA have increased annually. Research (80) has shown that aerobic exercise sessions exceeding 60 min can delay the progression of AGA, with the potential mechanisms including: ① The micronutrient concentrations necessary for HF proliferation are elevated through enhanced hemodynamic circulation and elevated blood oxygen saturation (81). Once adequate blood perfusion to HFs is established, the influx of nutrients and micronutrients may be enhanced. These elements can then be more effectively delivered into the follicular microenvironment, thereby potentially promoting HF proliferation under optimal conditions. ② Circulating testosterone concentrations exhibit an inverted-U relationship with exercise duration, whereby testosterone concentrations initially increase to a peak and then decline, ultimately dropping below baseline with prolonged activity (82–84). ③ Appropriate exercise modalities and intensities also can effectively mitigate stress, alleviate symptoms of anxiety and depression, and enhance sleep quality (85), all of which are factors that could potentially slow the progression of AGA.

However, further research indicates that individuals engaging in high-intensity physical exercises such as sprinting, resistance training, and weightlifting exhibit elevated androgen levels beyond normative ranges (86, 87). Arachidonic acid (AA) that is released during anaerobic exercise due to either micro-tears in muscle fibers (can activate phospholipase A2) or localized hypoxia (88) is rapidly metabolized by cyclooxygenase (COX) into prostaglandins (PGs) (89), which may induce or exacerbate AGA.

### 2.6 Ultraviolet radiation

One type of non-ionizing electromagnetic radiation in the electromagnetic spectrum is UVR, which comes mainly from the sun. The radiation wavelengths emitted by the sun include ultraviolet A (UVA, 320 ~ 400 nm), ultraviolet B (UVB, 280 ~ 320 nm), and ultraviolet C (UVC, <280 nm). Under normal conditions, human skin is typically exposed solely to ultraviolet B radiation (UVBR) and ultraviolet A radiation (UVAR), which can induce various dermatological pathophysiological processes (90): ① The critical regulatory factor for proliferation of HFs in AGA patients is the equilibrium of various PG subtypes within the scalp (91), which is susceptible to disruption by ultraviolet radiation exposure (92). ② UVR goes through the outer skin layer, damaging DNA and possibly affecting the growth of skin cells around hair follicles (93). ③ Prolonged exposure to UVR can induce a spectrum of immunomodulatory effects at both local and systemic levels. For example, UV exposure elevates the expression of pro-inflammatory cytokines such as Interleukin-1 alpha (IL-1α), IL-1β, IL-6, and TNF-α, promotes the generation of ROS, and mediates oxidative damage to cellular membranes, mitochondria, and DNA (94–97). Collectively, these alterations may contribute to the inhibition of HF proliferation. ④ Long-term UVR exposure induces photoaging of the scalp, manifesting as uneven epidermal thinning, dermal collagen degradation, and degeneration of elastic fibers (EFs) (98). Research elucidates that photoaging also exerts a notable impact on HFs (5): UVAR can lead to a reduction in HFSCs, transit-amplifying cells (TA cells), and melanocytes, which results in HF photoaging characterized by follicular miniaturization and an increase in graying. ⑤ The outermost layer of the hair shaft consists of keratin filament structures, which are highly responsive to environmental stimuli and undergo extensive structural alterations when exposed to UVR. Many lipids that may improve hair quality, such as ceramides, are observed in lower concentrations in UV-exposed hair (99), which may adversely affect hair health and could thereby contribute to hair loss.

### 2.7 Hairstyles

#### 2.7.1 Tight styling

In contemporary society, there is an increasing prevalence of various hairstyling techniques aimed at enhancing esthetic appeal. Attendant to that tension from various hairstyles with braids, locks, glue, tight buns, gels, nighttime tight hair wrapping, or the combined use of chemical relaxers with braids may increase the risk of hair loss (100). This condition is known as traction alopecia (TA). Although TA and AGA are distinct entities with different etiologies and clinical presentations, they can co-occur in the same individual. Their coexistence may exert synergistic effects, potentially leading to a collectively exacerbated progression of hair loss: The excessive pulling forces of some hairstyles cause mechanical damage to the hair follicles that induces an inflammatory response (101). Evidence suggests that the inflammatory response within HFs is closely associated with AGA (23).

#### 2.7.2 Color-treated styling

An essential part of color-treated styling is the use of hair dyes and bleaches, which can exert adverse effects at various levels, thus potentially worsening AGA progression: ① Bazargan A. et al. (102) found that a significant association exists between lighter hair colors. This finding may be attributed to individuals with lighter hair possessing reduced melanin, who may exhibit heightened vulnerability to oxidative damage and, as a result, an elevated risk of hair follicle shrinking and androgenetic alopecia (AGA) (102). ② Biochemical evidence indicates that carcinogenic substances present in hair dye and bleach can damage exposed cells, which may undergo apoptosis (103). In addition, hair dye and bleach preparations can cause significant hair shaft damage due to the dye molecules’ ability to penetrate the cuticle and reach the hair cortex (104). ③ Contact sensitizers (e.g., p-phenylenediamine, resorcinol, m-aminophenol, and p-aminophenol) were common in hair dye products (105), with hypersensitivity, immune response, and/or severe allergic reactions having been widely reported (104, 106). Studies have shown that AGA patients often have inflammatory cell infiltration around the hair follicles (107).

#### 2.7.3 Heat styling

Heat styling (such as with a hair dryer, straightener, or curling iron) does not directly cause AGA, but it can cause significant damage to the hair and scalp, which may increase the burden on HFs and potentially accelerate symptoms of AGA patients: After repeated shampooing and drying, definite damage to the hair cuticle was evident on scanning electron microscopy (SEM) examination. Hair damage due to heat can be found on the surface, cuticle layers, and possibly the cell membrane complex (CMC) (108).

## 3 Pathological mechanisms of AGA

The precise pathological mechanisms underlying AGA remain incompletely elucidated. Through an analysis of the pathological mechanisms related to the lifestyle-induced AGA and current research hotspots (63), it has been found that the key pathogenic mechanisms of AGA are primarily concentrated within the following pathways:

### 3.1 Wnt/β-catenin pathway

**Figure 2:**
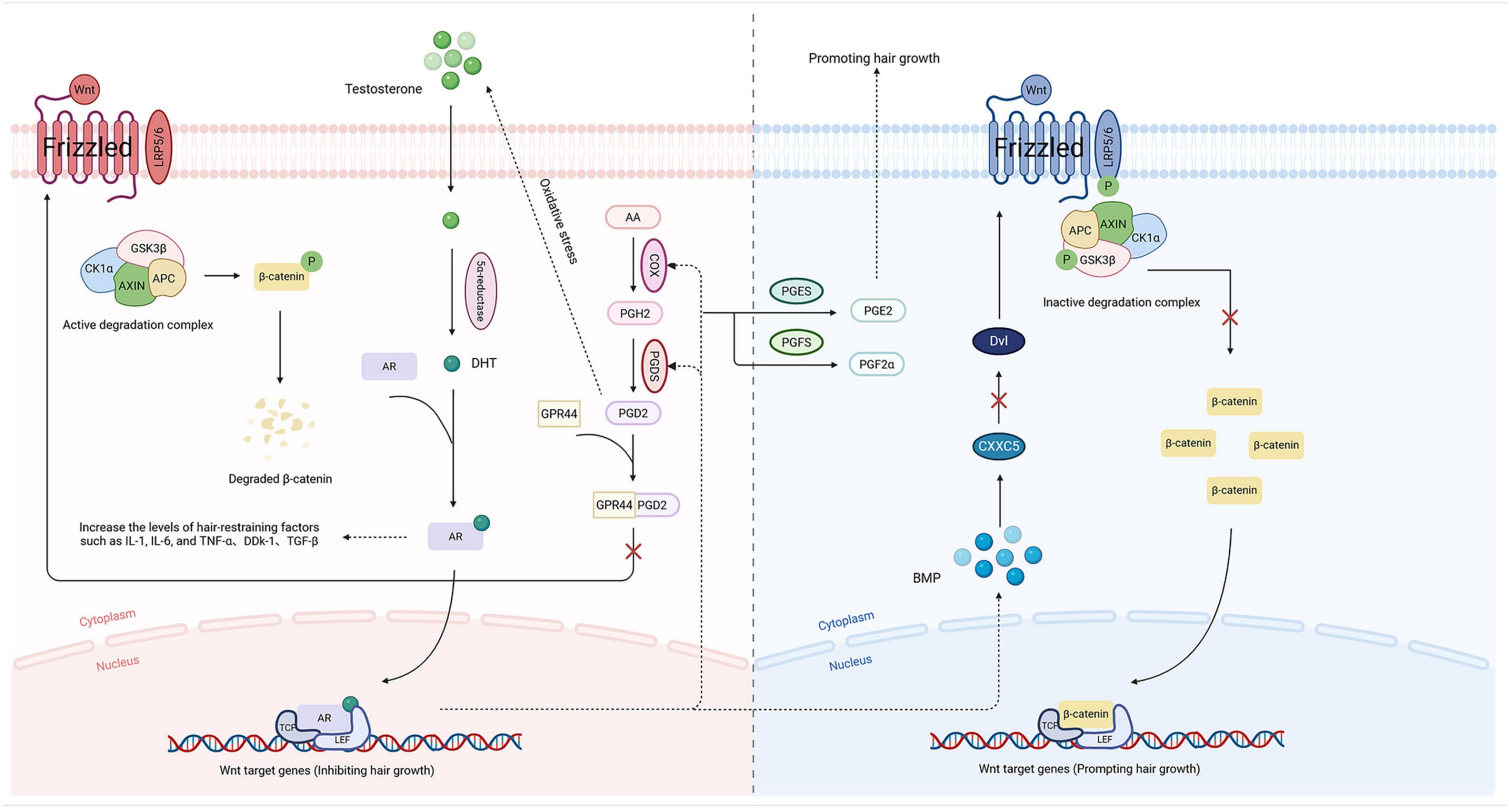
The Wnt/β-catenin pathway of AGA (Low-density lipoprotein receptor-related protein 5/6, LRP5/6; Prostaglandin D synthase, PGDS; Prostaglandin E synthase, PGES; Prostaglandin F synthase, PGFS; Prostaglandin H2, PGH2; G protein-coupled receptor 44, GPR44; CXXC type zinc finger protein 5, CXXC5). The figure has been designed using resources from BioRender.com.

The Wnt/β-catenin pathway, which plays a key role in embryonic development and adult tissue homeostasis, is implicated in the pathogenesis of numerous diseases, including AGA, bone disorders, neurodegenerative diseases, and tumors, when dysregulated (109). During the transduction process of the Wnt/β-catenin pathway, Wnt proteins are combined with Frizzled receptors and low-density lipoprotein receptor-related proteins (LRP) co-receptors, resulting in the inactivation of glycogen synthase kinase-3 beta (GSK-3β, a kinase that initiates its ubiquitination and proteasomal degradation through the phosphorylation of β-catenin) of the degradation complex composed predominantly of axis inhibitor (Axin), casein kinase 1 alpha (CK1α), adenomatous polyposis coli (APC), and GSK-3β (110). Therefore, the inactivation of GSK-3β stabilizes cytoplasmic β-catenin. Alternatively, activated disheveled (Dvl) combines with Axin to inhibit GSK-3β activity via phosphorylation, allowing cytosolic β-catenin to evade degradation (111). Stable β-catenin activates a transcriptional cascade of genes that promote HF proliferation and hair growth when it binds to T-cell factor (TCF)/Lymphoid Enhancer Factor (LEF) transcription factors in the nucleus (109) (Figure 2).

### 3.2 DHT–PGD2 loop

PGs, lipid compounds derived from AA metabolism, are widely distributed in tissues and bodily fluids and play roles in numerous physiological processes and inflammatory responses, including cell proliferation, differentiation, and apoptosis (112). PGD2, a signaling molecule derived from AA via COX and prostaglandin D synthase (PGDS) (89), binds to the G protein-coupled receptor 44 (GPR44), resulting in potent suppression of the Wnt/β-catenin pathway and antagonism of pro-hair growth mediators such as PGE2 and PGF2α (91), with a potential consequence being the impairment of HF proliferation.

The testosterone levels, which are converted to DHT with increased AR affinity under the action of 5α-R, may be indirectly elevated due to oxidative stress induced by PGD2 (113). The DHT-AR complexes translocate into the nucleus and competitively bind to TCF/LEF transcription factors, thereby preventing β-catenin from initiating the transcription of genes responsible for hair growth. This process also can inhibit proliferation of HFs by activating the BMP/Smad pathway, which leads to: ① upregulation of ligands like bone morphogenetic protein 4 (BMP4), which activates CXXC type zinc finger protein 5 (CXXC5) to inhibit Dvl; ② insufficient proliferation of HFSCs, triggering follicular miniaturization; and ③ elevated expression of hair growth suppressors such as TNF-α, dickkopf-related protein 1 (DKK-1), and IL-6, ultimately leading to the inhibition of HF proliferation (114–117) (Figure 2).

### 3.3 Oxidative stress and inflammation

One of the fundamental requirements of life is redox homeostasis, which permeates the entire process of metabolism throughout the lifespan (118). Oxidative stress refers to the disruption of the redox homeostasis between pro-oxidants and antioxidants. Oxidative stress refers to the disruption of the oxidant-antioxidant balance (118). Once this balance is broken, the dermal papilla cells (DPCs) that regulate the formation, proliferation, and cyclicity of HFs will show obvious oxidative stress characteristics, such as mitochondrial dysfunction, cell apoptosis, and perifollicular inflammation, which is associated with HF miniaturization, a process that can culminate in hair loss (26, 119). Furthermore, the activation of the TGF-β1/Smad pathway by ROS leads to the upregulation of critical apoptotic factors for hair growth, like TGF-β1 and transforming growth factor beta 2 (TGF-β2), thereby triggering senescence and damage of DPCs and ultimately contributing to AGA (120–122). Numerous studies support the interdependence of oxidative stress and inflammation (123, 124): ① Inflammatory cells in the scalp can release substantial amounts of ROS, which exacerbate oxidative damage around the microenvironment of HFs; ② ROS also can exacerbate inflammatory responses, inducing excessive secretion of hair growth inhibitory factors such as IL-1, IL-6, TNF-α, and DKK-1. This bidirectional relationship may induce or exacerbate AGA due to the persistent inflammatory milieu surrounding HFs.

### 3.4 Microecological dysbiosis

**Figure 3:**
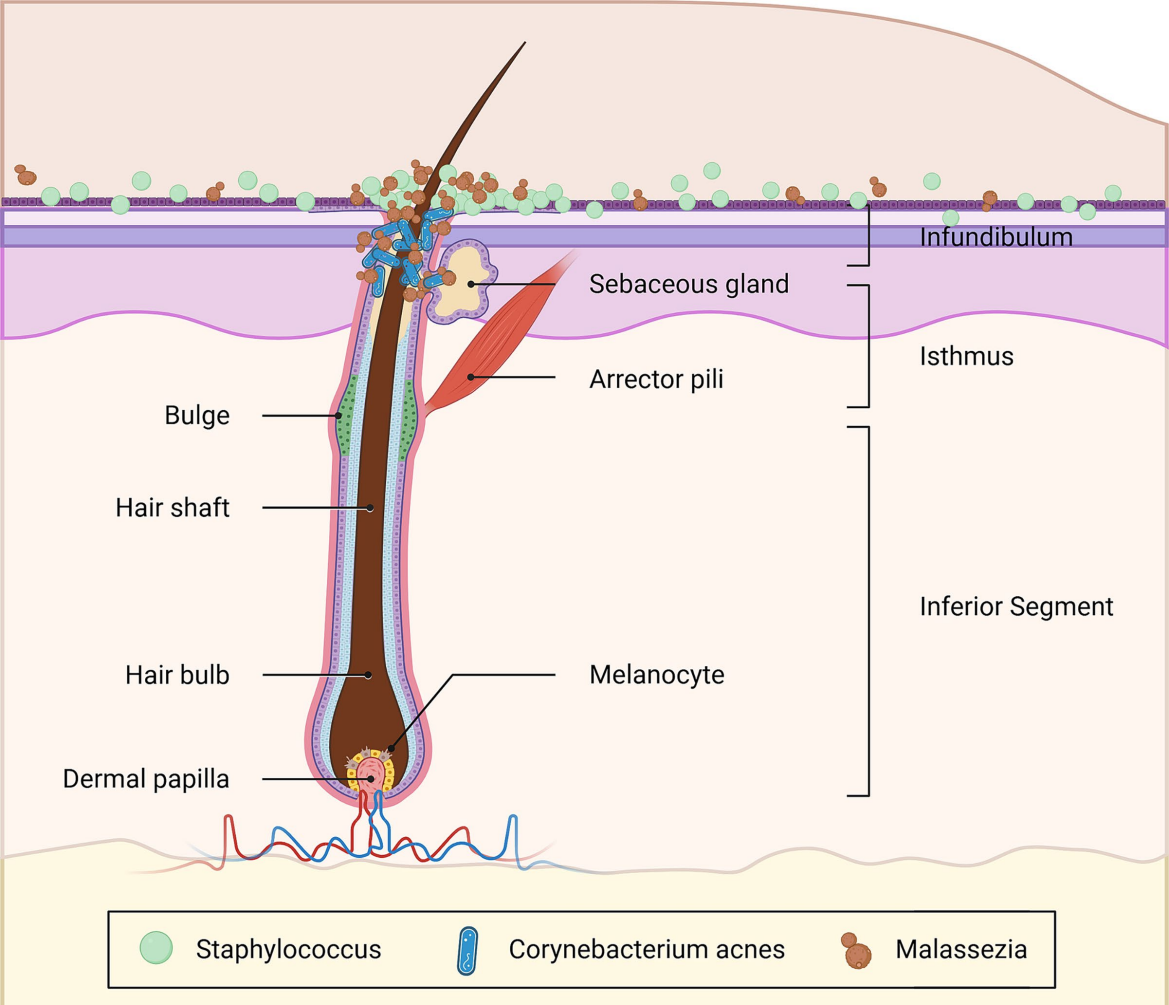
The distribution of microbes around HFs. The figure has been designed using resources from BioRender.com after referring to “The role of the microbiome in scalp hair follicle biology and disease”.

Polak-Witka K. et al. (125) based on metagenomics confirmed that humans’ HFs constitute a complex microecosystem that is inhabited by diverse microbial communities, predominantly hosting symbiotic microbiota such as *Malassezia*, *Cutibacterium*, *Staphylococcus*, etc. Research indicates that approximately 50% of AGA patients’ skin biopsies exhibit a significant infiltration of mononuclear cells (MNCs) and lymphocytes (Lymphs) within the upper third of HFs hosting numerous microorganisms (125), as shown in Figure 3. The excessive proliferation of *Malassezia*, a key contributor to skin homeostasis, triggers a cascade of events: ① it stimulates KCs to secrete cytokines (such as TNF-α, IL-1β, and IL-6), thereby elevating peribulbar cytokine levels and exacerbating follicular inflammation (30); ② it directly compromises the integrity of HFs via its proteolytic enzymatic activity (30); ③ it generates a substantial amount of ROS that contributes to oxidative stress (126).

The study by Ho BS et al. reported a higher detection rate of *Cutibacterium acnes* in miniaturization of HFs of AGA patients (43.3%) compared to healthy follicles (7.79%) (127). *Cutibacterium acnes* possesses a genome that encodes multiple enzymes required for a complete porphyrin metabolic pathway. Porphyrin, the metabolic end product, incites localized follicular inflammation by inducing oxidative stress and promoting the secretion of multiple pro-inflammatory cytokines (128). The clinical presentation of AGA may represent the integrated outcome of a synergistic interplay among multiple pathological mechanisms, including microecological dysbiosis, oxidative stress, and inflammatory responses.

## 4 Recommendation

In summary, the following lifestyles are recommended for AGA patients in daily life: ① A beneficial dietary strategy involves maintaining a low-fat diet and a regular dietary habit to avoid extremes of starvation or overeating. Furthermore, evidence suggests that limiting excessive caffeine intake is also important for reducing the risk of developing AGA (40). ② Individuals are encouraged to avoid tobacco use. For non-smokers, minimizing exposure to secondhand smoke is equally important for health. ③ For patients who drink alcohol, it is recommended that reducing alcohol consumption or quitting drinking be included as part of their management plan. ④ Individuals are encouraged to adhere to a regular, sufficient sleep pattern and foster emotional stability as part of their management plan. ⑤ For patients with AGA, prolonged engagement in high-intensity anaerobic exercise may potentially accelerate the progression of HF miniaturization and hair loss, while too short aerobic activity fails to produce therapeutic benefits. Therefore, it is recommended that AGA patients with normal cardiopulmonary function engage in aerobic exercise exceeding 60 min to optimize clinical outcomes (80). ⑥ It is advisable to practice consistent sun protection as a routine measure, with special consideration given to shielding the scalp from UVR. ⑦ The following styling practices can be beneficial in reducing tension on hair follicles: loose, low-hanging ponytails and buns; wigs worn with satin cap; natural/unprocessed hair (129).

## 5 Discussion

The review indicates that patients with AGA may be susceptible to various lifestyles, such as bad dietary habits, smoking, alcohol consumption, sleep disturbances, psychological stress, UVR, excessive anaerobic exercise, and hairstyle. One limitation that should be noted in the interpretation of the review is the limited clinical evidence regarding the impact of lifestyles on AGA. Therefore, it is imperative to conduct further clinical trials or epidemiological studies utilizing *in vivo* or *in vitro* experiments to elucidate the precise molecular mechanisms underlying AGA, which may also inform future recommendations for lifestyles in AGA patients.
